# Endoplasmosis and exoplasmosis: the evolutionary principles underlying endocytosis, exocytosis, and vesicular transport

**DOI:** 10.1007/s10354-016-0453-2

**Published:** 2016-05-11

**Authors:** Johannes A. Schmid

**Affiliations:** Center for Physiology and Pharmacology, Department of Vascular Biology and Thrombosis Research, Medical University Vienna, Schwarzspanierstraße 17, 1090 Vienna, Austria

**Keywords:** Lipid Bilayers, Membrane Fusion, Eukaryotic Cells, Extracellular Space, Membranes, Endocytosis, Exocytosis, Lipiddoppelschicht, Membranfusion, Eukaryotische Zellen, Extrazellulärraum, Membrane, Endozytose, Exozytose

## Abstract

Eukaryotic cells are characterized by a multicompartmental structure with a variety of organelles. Vesicular transport between these compartments requires membrane fusion events. Based on a membrane topology view, we conclude that there are two basic mechanisms of membrane fusion, namely where the membranes first come in contact with the cis-side (the plasmatic phase of the lipid bilayer) or with the trans-side (the extra-plasmatic face). We propose to designate trans-membrane fusion processes as “endoplasmosis” as they lead to uptake of a compartment into the plasmatic phase. Vice versa we suggest the term “exoplasmosis” (as already suggested in a 1964 publication) for cis-membrane fusion events, where the interior of a vesicle is released to an extraplasmatic environment (the extracellular space or the lumen of a compartment). This concept is supported by the fact that all cis- and all trans-membrane fusions, respectively, exhibit noticeable similarities implying that they evolved from two functionally different mechanisms.

## Zusammenfassung

Eukaryotische Zellen besitzen eine Multi-Kompartiment-Struktur mit unterschiedlichen Organellen. Vesikulärer Transport zwischen den Kompartimenten erfordert Membranfusionsprozesse. Bei Betrachtung aus Sicht der Membrantopologie kann man schließen, dass es 2 grundsätzliche Mechanismen der Membranfusion gibt: einerseits solche, bei denen die Membranen zuerst mit der cis-Seite (der plasmatischen Seite der Membrandoppelschicht) in Kontakt treten, und andererseits solche, bei denen sich die trans-Seiten zuerst berühren (die extraplasmatischen Seiten). Der Autor schlägt die Bezeichnung „Endoplasmose“ für alle trans-Membranfusionen vor, da sie zu einer Aufnahme eines Kompartiments in die plasmatische Phase führen, und vice versa die Bezeichnung „Exoplasmose“ (wie bereits 1964 in einer Publikation vorgeschlagen) für alle cis-Membranfusionen, wo das Innere eines Vesikels in einen extraplasmatischen Raum entladen wird (den Extrazellulärraum oder das Lumen eines Kompartiments). Dieses Konzept wird durch die Tatsache unterstützt, dass alle cis- bzw. alle trans-Membranfusionen bemerkenswerte Ähnlichkeiten zeigen, was darauf hindeutet, dass sie aus 2 funktionell unterschiedlichen Mechanismen entstanden sind.

## Introduction

While Darwin developed his theory of evolution for the macrobiological world, it is becoming increasingly evident that the general concept of evolution also applies to the cellular and subcellular world. In general, a central characteristic of all known organisms is their compartmentalisation, their delimitation from the surrounding environment. This compartmentalisation is a prerequisite for the generation and maintenance of electrochemical gradients that are essential for the energy processes of life. In contrast to prokaryotes, which consist in principle of only one “reaction compartment”, eukaryotes are characterised by a multicompartmental structure of various organelles within one common compartment. One of the crucial questions in that respect is how the intracellular organelles of eukaryotes evolved. The cytoplasmic membrane, which functions as the ultimate border of a cell, separates a cytoplasmic from an extra-cytoplasmic phase, or in other terms a plasmatic from a nonplasmatic sphere. Due to the multicompartmental organisation of eukaryotes, the cell contains internal nonplasmatic domains, namely all the compartments that are enclosed by a single lipid bilayer, like the endoplasmic reticulum (ER), the Golgi, endosomes or lysosomes.

**Fig. 1:**
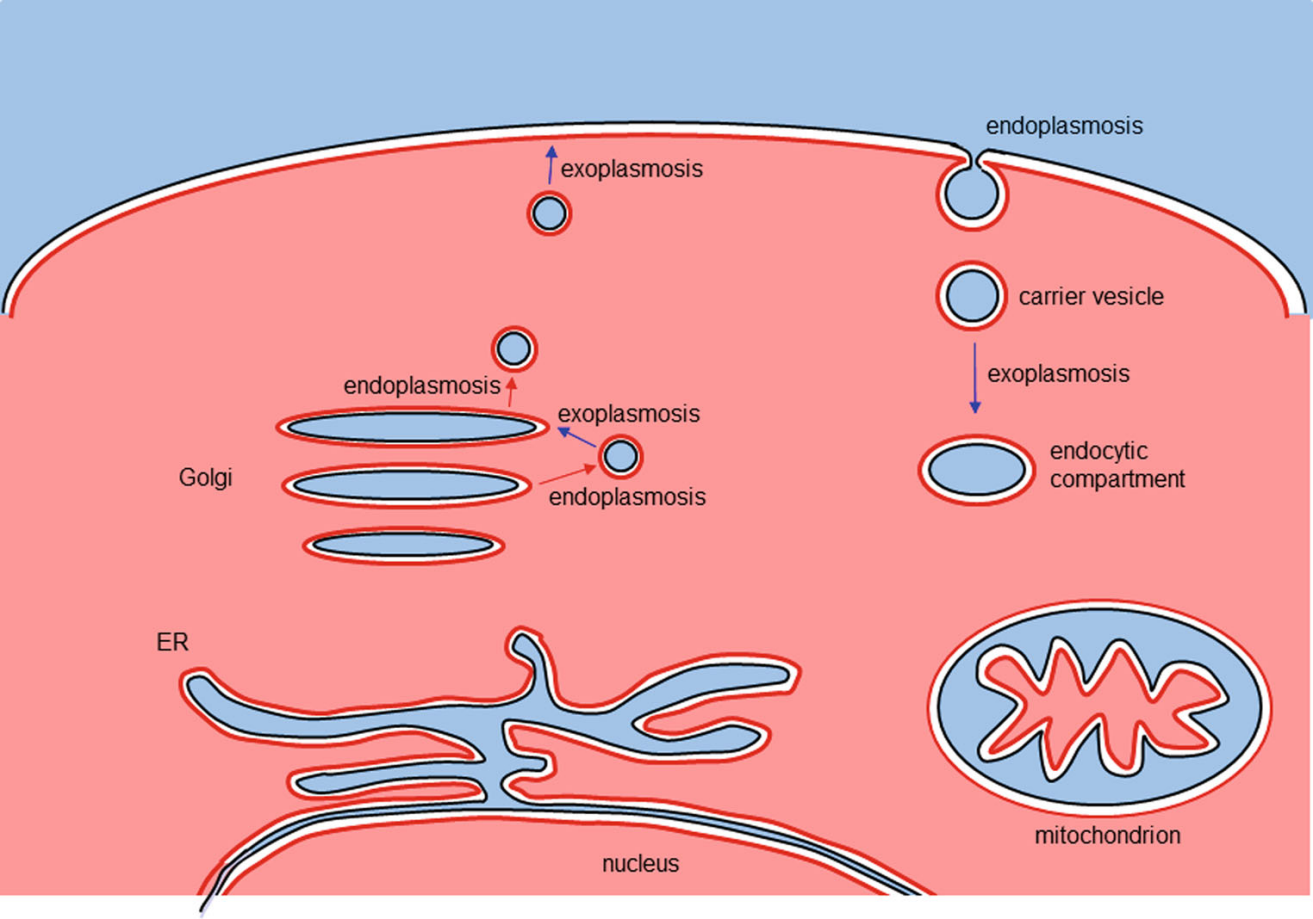
General scheme of the compartmental structure of eukaryotic cells and vesicular transport processes. Examples of different intracellular organelles are outlined. Plasmatic domains are in *red* and plasmatic membrane surfaces in *red thick lines*, nonplasmatic spheres are in *blue* and extraplasmatic membrane surfaces in *black lines*. Endoplasmosis (the budding of vesicles into a plasmatic environment, due to fusion of extraplasmatic membrane surfaces) is indicated by *red arrows*; exoplasmosis (release of vesicle contents into nonplasmatic domains after cis-membrane fusion) is outlined by *blue arrows*

Organelles that are enclosed by two lipid bilayers like chloroplasts or mitochondria exhibit a nonplasmatic intermembrane space, but their internal lumen is defined as plasmatic sphere comparable to the cytoplasm ([2, 3]; Fig. 1). This view is in agreement with the commonly accepted hypothesis that they were taken up from the extracellular environment by ancient pre-eukaryotes as endosymbionts [4–7]. Another organelle enclosed by two membranes is the nucleus, which is thought to have evolved from the ER [8] and which contains a plasmatic lumen that is linked to the cytosol via nuclear pore complexes that are permeable for ions and small proteins.

## An evolutionary perspective of vesicular transport

**Fig. 2:**
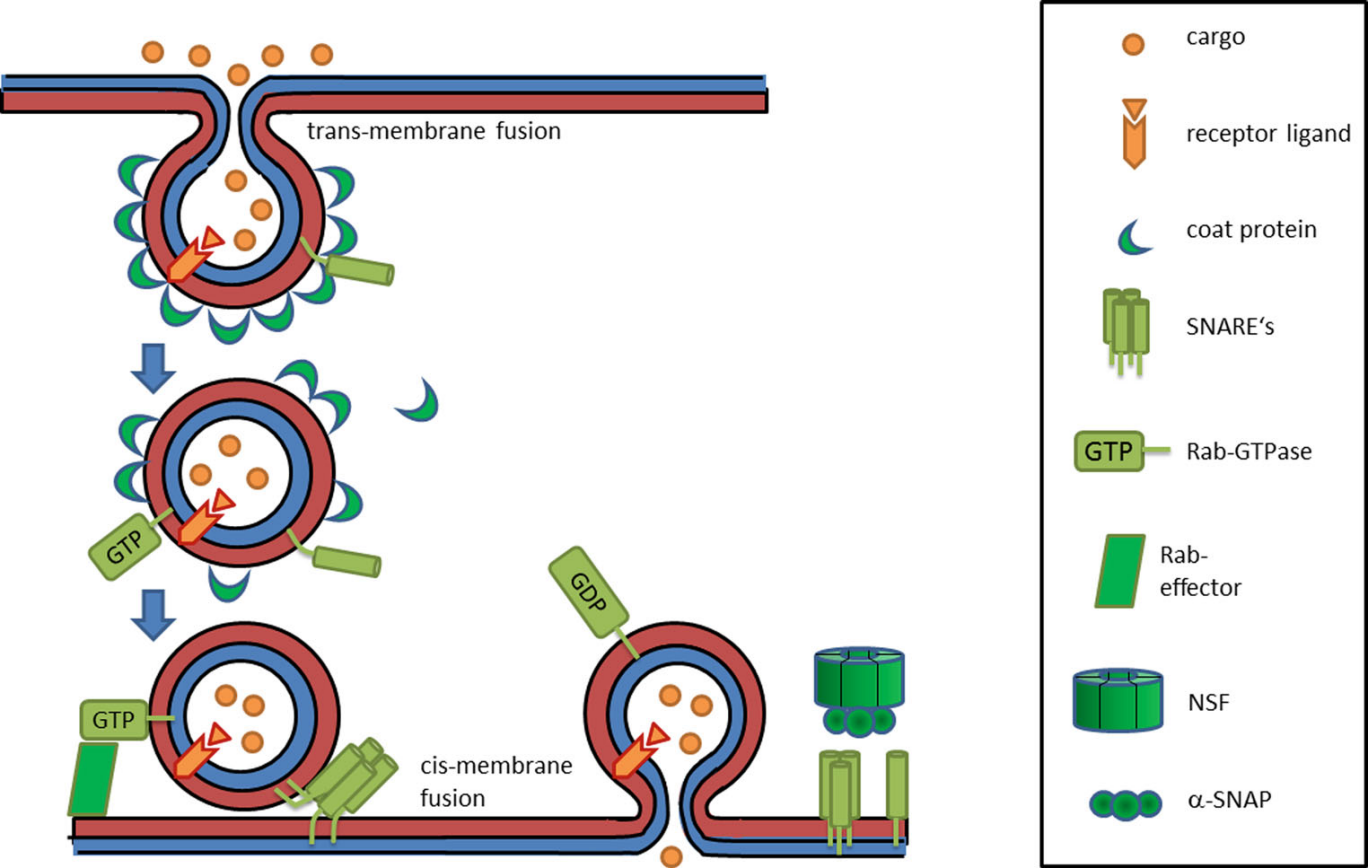
General mechanism of trans- and cis-membrane fusion events. The extraplasmatic sides of membranes (trans-sides) are shown in *blue*, the plasmatic faces in *red* (cis-sides). The *upper part* shows the budding of a vesicle into the plasmatic phase (endoplasmosis), characterized by the assembly of coat complexes and the segregation of fluid phase cargo or receptor–ligand complexes by a fission process, which is actually driven by a trans-membrane fusion event. The *lower part* shows the fusion of a vesicle with a target membrane (exoplasmosis). Rab GTPases, their effector proteins, SNARE proteins and accessory factors (such as NSF or SNAPs) are involved in these cis-membrane fusion processes

An important question in the evolution of eukaryotic cells is how the compartments arose, which are enclosed by a single membrane. The common explanation is that they were originally formed by invagination and internalization of the cytoplasmic membrane [9]. Interestingly, these first endomembranes might have had rather secretory functions than features of current endocytic compartments given that certain GTPases of the secretory pathway seem to have evolved before those of the endocytic pathway [10]. Regardless of how these first endomembranes formed and whether they were more endocytic or more secretory in nature, they can be regarded as “internalized extracellular space”. From this point of view, it is a provoking but intriguing aspect to regard all nonplasmatic compartments within the cell as spheres, which are in some way functionally outside of the cell. The ER for instance can be regarded as first outstation of the cell in the course of secretion, which might have evolved as an internalised and specialised part of the cytoplasmic membrane [9]**.** Molecules that are co- or posttranslationally transported into the lumen of the ER are therefore equivalent to proteins secreted from prokaryotes. Strikingly, the ER and other organelles are not synthesised *de novo *in developing cells, but handed over from parental cells (“omnis membrana e membrana” [9]), supporting the notion that the compartmental organisation of eukaryotes is not encoded in the genome, but directly inherited. This notion also suggests that the eukaryotic ancestor cells must have had biological membranes [11]. The structure of eukaryotic cells with plasmatic and nonplasmatic spheres implies that the lipid bilayers, which form the membrane of organelles or the boundary between the cytoplasm and the extracellular environment, exhibit a plasmatic (or *cis-*) and an extraplasmatic (or *trans-*) face [3]. Endocytosis and exocytosis, as well as the membrane traffic within the cell require fusion and budding events that are all based on membrane fusion, where either the plasmatic or the extraplasmatic faces of two lipid bilayers first come in contact. These processes can be designated as cis- or trans-membrane fusion events, respectively. As a consequence of this view, we propose to designate all trans-membrane fusion processes (where the extraplasmatic membrane surfaces first come in contact) with the term “*endoplasmosis*”, meaning an uptake of a vesicle or membrane compartment into a plasmatic phase. Vice versa, all cis-membrane fusion processes can be termed as “*exoplasmosis*” meaning that a compartment fuses with a membrane with the two cis-sides coming into contact first, resulting in the release of the cargo into a nonplasmatic phase (Figs. 1 and 2). It has to be stated that the term “exoplasmosis” was used in an early publication on leukocyte degranulation in 1964 [1], but has not been applied systematically since then. Using these terms has the advantage of emphasizing conceptual similarities between different processes of endocytosis, exocytosis and vesicular transport as they stress the two underlying basic principles from a cell topology perspective. Endoplasmosis then subsumes processes such as the first step of endocytosis, or the budding and formation of carrier vesicles, for instance from ER, Golgi stacks or endosomes. Exoplasmosis would be the superordinate term for fusion of secretory vesicles with the plasma membrane, the fusion of carrier vesicles with target membranes and all other cis-membrane fusion events, where vesicles functionally leave the plasmatic sphere.

This concept is in line with the fact that there are many similarities between different forms of endoplasmosis or exoplasmosis, meaning trans- and cis-membrane fusion events, respectively. A common feature of endoplasmosis is the requirement for coat proteins at the plasmatic face of the membrane (Fig. 2). For endocytic internalisation, these coats can consist of clathrin [12] or caveolin [13, 14]. Furthermore, clathrin-coated areas can also be detected on the trans-Golgi network [12] and under certain circumstances also on sorting endosomes [15]. Along the exocytic route, the budding and formation of carrier vesicles from ER *en route* to the intermediate compartment requires COP-II (coat protein complex II) containing coats [16]. Finally, recycling of vesicles to the ER, as well as transport within the Golgi is dependent on COP-I [17–20]. Some of these coat proteins that are characteristic for the exocytic pathway (COP-I and ARF [ADP-ribosylation factor]) were also detected on endosomal membranes [21].

**Fig. 3:**
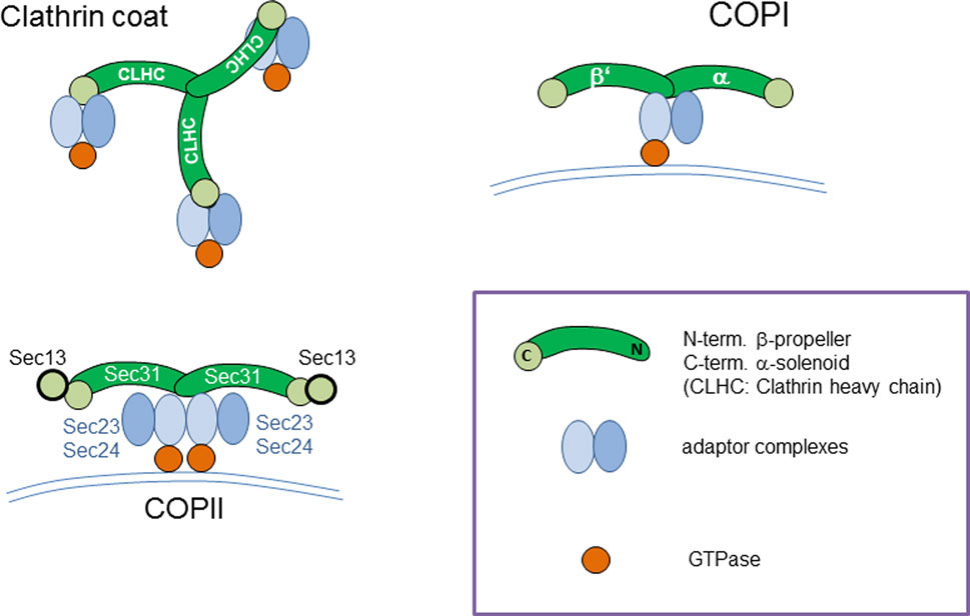
Structural similarities between different coat complexes: Components of the clathrin, COPI and COPII coats are shown in a structural schematic view. Coat structures are shown in *green*; adaptor complexes in *blue* and GTPases in *red*. (Adapted from [6, 16])

**Fig. 4:**
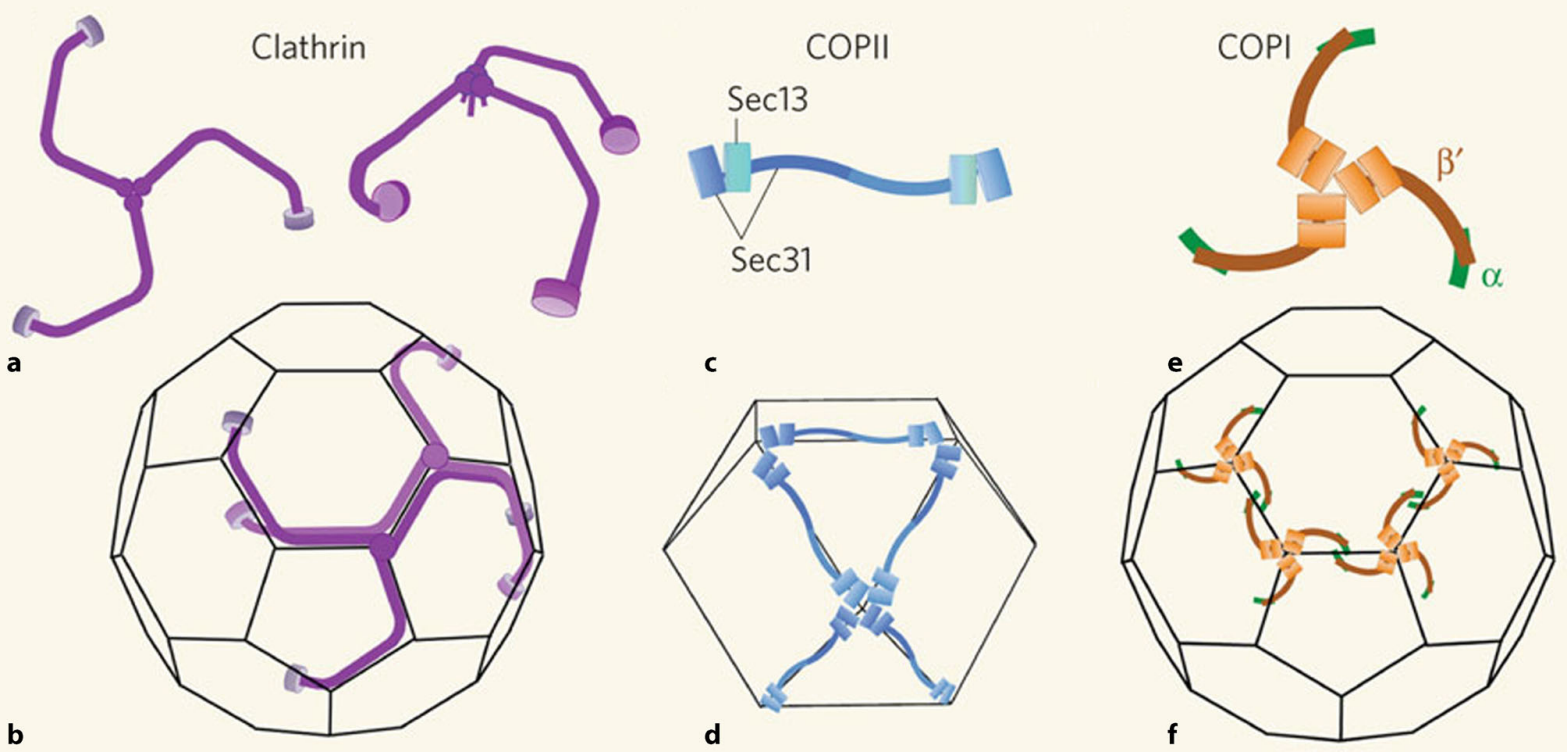
Structural conservation between distinct vesicular coat complexes occurring at the cell surface, ER or Golgi membranes. (Reprinted with permission from Macmillan Publishers Ltd [27])

Thus, all these examples of trans-membrane fusion events, which can be subsumed as endoplasmosis events, are characterised by the formation of coat structures at the cytosolic face of the membrane [22, 23], which seems to be important for generating a curvature of the membrane preceding the budding process [24–26]. This view of a general principle underlying all these trans-membrane fusions events is supported by the fact that coat subunits from functionally distinct coats such as clathrin, COPI and COPII coats exhibit noticeable structural similarities with β‑propeller and α‑solenoid elements in a specific common arrangement (Fig. 3; reviewed in [6]). Furthermore, the proteins involved bind to each other in a way that leads to a very similar molecular architecture of the coat complex on the membrane as illustrated in Fig. 4 (reviewed in [27]) pointing at a common evolutionary origin of trans-membrane fusion events.

**Fig. 5:**
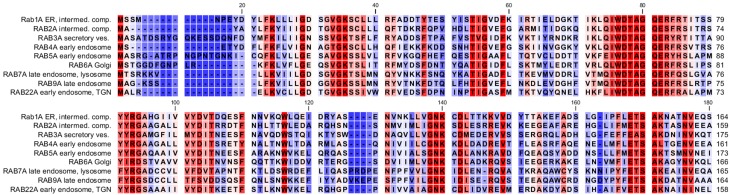
Alignment of human Rab GTPases and allocation to their main intracellular organelles: homologous amino acids are shown in different shades of *red*. The main localizations of the GTPases were obtained from reference [29]

**Fig. 6:**
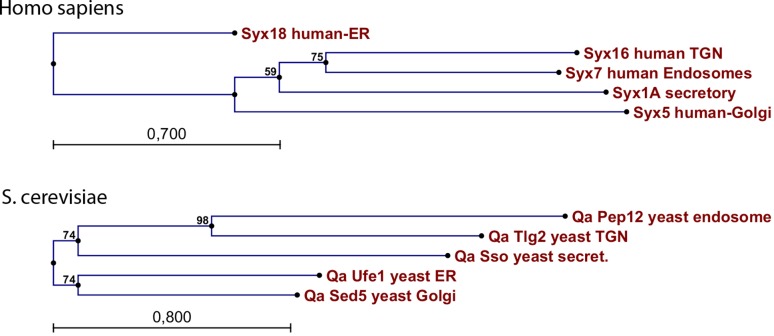
Phylogenetic analysis of Qa-type SNARE proteins: *Upper part*: Human syntaxins specific for various organelles are compared; *lower part*: the respective Qa proteins from S. cerevisiae are shown

On the other hand, cis-membrane fusion processes from clearly different organelles also show striking mechanistic similarities as well as sequence homologies of the proteins that are involved therein. This holds true for fusion of carrier vesicles with target membranes in Golgi transport, fusion of secretory granules with the cytoplasmic membrane or endosome fusion, which are all dependent on certain fusion factors such as NSF (N-ethylmaleimide sensitive factor), SNAPs (soluble NSF attachment proteins), SNAREs (SNAP receptors) and Rab family GTPases (guanosine triphosphatases) (Fig. 2; [28–39]). The evolutionary coherence of all these distinct membrane fusion processes is well demonstrated by the strong conservation and high degree of homology of Rab GTPases, which are important regulators of cis-membrane fusions at completely different organelles and locations within the cell (Fig. 5). Significant homologies are also found for other fusion factors such as SNARE proteins—and again their functional role seems to be the same for all the different cis-membrane fusion processes at distinct organelles within the cell. Phylogenetic analysis of the Qa family of human SNARE proteins (syntaxins) for various endomembranes reveals clear links, with syntaxins located at endosomes being closely related to syntaxins of the trans-Golgi network and slightly more distant to those of secretory vesicles. A very similar phylogenetic tree is observed for the Qa proteins of yeast (Fig. 6). However, in the latter case the proteins are more related to each other suggesting that higher order organisms developed a higher diversity of these fusion factors. While these sequence analyses clearly point at a common evolutionary origin of the different cis-membrane fusion processes, it is also clear that in current organisms and cells a high level of specificity is observed for cis-membrane fusion events of distinct intracellular compartments. This specificity is crucial for maintaining the functional integrity of the organelles, an ordered progress of vesicular transport and the “identity” of the membrane compartments. The available data indicate a model, in which all cis-membrane fusions go back to an ancient process of “exoplasmosis”, which was then altered by evolutionary diversification forming specific organelles and well-controlled fusion processes.

## Conclusion

Based on the similarities between different membrane fusion processes, we suggest that there are two generally distinct mechanisms of membrane fusion, namely endoplasmosis (trans-membrane fusion) and exoplasmosis (cis-membrane fusion), which are the mechanistic ancestors of all the cellular membrane fusion events. These two basic principles of membrane fusion might be the origin for the evolution of the eukaryotic endomembrane system, which developed its complexity by diversification of the components, finally defining the identities of intracellular compartments and regulating the membrane traffic between them [40–42].

## List of abbreviations

ARF: ADP-ribosylation factor
COP: coat protein complex
ER: endoplasmic reticulum
GTPase: guanosine triphosphatase
NSF: N-ethylmaleimide sensitive factor
SNAP: soluble NSF attachment protein
SNARE: SNAP receptor
